# Salivary IL-17A, IL-17F, and TNF-*α* Are Associated with Disease Advancement in Patients with Oral and Oropharyngeal Cancer

**DOI:** 10.1155/2020/3928504

**Published:** 2020-08-13

**Authors:** Kinga Zielińska, Bożenna Karczmarek-Borowska, Konrad Kwaśniak, Justyna Czarnik-Kwaśniak, Aleksandra Ludwin, Bogumił Lewandowski, Jacek Tabarkiewicz

**Affiliations:** ^1^Mrukmed Medical Centre, Rzeszów 35-922, Poland; ^2^Department of Clinical Oncology, Subcarpatian Oncology Centre of The Specialist Hospital in Rzeszow No. 1, 35-055 Rzeszow, Poland; ^3^Department of Clinical Oncology, Institute of Medicine, College for Medical Sciences of University of Rzeszow, 35-959 Rzeszow, Poland; ^4^Laboratory for Translational Research in Medicine, Centre for Innovative Research in Medical and Natural Sciences, College for Medical Sciences of University of Rzeszow, 35-310 Rzeszow, Poland; ^5^Department of Immunology, Institute of Medicine, College for Medical Sciences of University of Rzeszow, 35-959 Rzeszow, Poland; ^6^Department of Oral and Maxillofacial Surgery, Institute of Medicine, College for Medical Sciences of University of Rzeszow, 35-959 Rzeszow, Poland

## Abstract

Delayed diagnosis of oral cavity and oropharyngeal cancer is associated with a poor prognosis. Despite progress in systemic therapy and radiotherapy, there has only been a slight improvement in the five-year survival rate. A non-invasive diagnostic method that consists of an assessment of specific proteins in saliva samples may significantly facilitate assessment of treatment results in patients diagnosed with oral and oropharyngeal cancer. The aim of this study was to assess the levels of IL-17 and TNF-*α* in the saliva of patients with oral and oropharyngeal cancer. The study was conducted prior to treatment in patients hospitalized in the Frederic Chopin Provincial Specialist Hospital No. 1 in Rzeszów, Poland. Saliva samples were collected from subjects on an empty stomach. Cytokine concentrations in the saliva were measured with ELISA and Luminex Multiplex Assays. The higher salivary concentrations of IL-17A, IL-17F, and TNF-*α* were significantly associated with disease advancement. Lower levels of IL-17A were associated with colonization of the oral cavity with aerobic bacteria. On the other hand, higher concentration of TNF-*α* was observed in patients with positive aerobic culture of oral swabs. Our results suggest that IL-17A, IL-17F, and TNF-*α* measured in the saliva may be a potential biomarker for cancer of the oral cavity and oropharynx.

## 1. Introduction

A poor prognosis of oral cavity and oropharyngeal cancer is associated with delayed diagnosis, and there is a lack of reliable biomarkers for these diseases. Each year, over 350,000 new cases are diagnosed and almost 180,000 deaths are reported due to oral cancer, making this disease a significant worldwide health problem [1]. Delayed cancer diagnosis is a factor contributing to the low survival rate. Patients with specific risk factors for oral cancer, such as smoking, excessive alcohol consumption, and human papillomavirus infection, should regularly assess their oral condition as a part of preventive examinations. Oral squamous cell carcinoma (OSCC) is the most commonly diagnosed head and neck cancer and its incidence has increased in recent decades. OSCC prevalence among women and young or middle age men has also been increasing [2]. Despite the progress in systemic therapy and radiotherapy, there has been only a slight improvement in the five-year survival rate. In the past, approaches using saliva samples in the diagnostic process of an early detection of oral and oropharyngeal cancer were made. Elevated concentrations of various active compounds in saliva samples indicate the possibility of using them as potential biomarkers [3]. There are many potential uses for saliva for diagnosis and monitoring of diseases due to the rapid and non-invasive accumulation process. Much research has been dedicated to the role of saliva in the diagnosis of oral cancer. A non-invasive diagnostic method consisting of an assessment of specific proteins in a saliva sample may be used in screening tests. It may significantly facilitate the assessment of the results of the treatment in patients diagnosed with oral and oropharyngeal cancer.

Inflammation plays a crucial role in various stages of cancer, and cytokines, as a group of host immune system products, are involved. Within the tumor, there are cytokines that originate from tumor-infiltrating immune cells and from tumor cells. Cytokine concentrations are maintained at a specific level at a given time. Numerous studies have been conducted to determine the role of cytokines as potential tumor markers. Cytokines were assessed in the serum, saliva, and tumor tissue, and some results indicate the possibility of determining interleukin levels in order to differentiate neoplastic lesions from benign [4–6]. This also applies to the development of oral squamous cell carcinoma [7–9]. Many studies have shown the potential of using proinflammatory cytokines as markers of oral cancer by determining their concentration in serum or in cancerous tissue [5, 10, 11]. In a study by Korostoff et al., elevated levels of TNF-*α* in the saliva were found in patients diagnosed with tongue cancer compared to the control group [12]. Juretić et al. showed significant differences in the concentration of TNF-*α* in saliva in a group diagnosed with oral cancer compared to the control group [7]. Similarly, Rhodus et al. found significant differences in salivary concentration of TNF-*α* in squamous cell carcinoma, oral premalignant lesion, and the control group [13]. On the other hand, Brailo et al. did not observe differences in TNF-*α* concentration in the saliva among groups with either cancer, leukoplakia, or in healthy people [8].

The IL-17 family consists of six cytokines (from IL-17A to IL-17F), which are produced mainly by Th17 cells. IL-17 plays an active role in autoimmune diseases and rejection of transplanted organs and has anticancer effects [14]. On the other hand, Th17-related cytokines can initiate tumor progression, angiogenesis, and metastasis. IL-17A has been found in many types of cancer, e.g., ovarian cancer and pancreatic cancer [15, 16]. In hepatocellular carcinoma and fibrosarcoma, the presence of IL-17A increases tumor progression through the development of angiogenesis and metastasis [17, 18]. However, high IL-17 expression is associated with better progression-free survival in advanced ovarian cancer [19]. Animal models indicate that overexpression of IL-17 in tumor cells suppresses tumor progression through increased antitumor immunity in immunocompetent mice [20]. Th17 cells and IL-17 levels are increased in the peripheral blood of patients with squamous cell carcinoma of the head and neck [21].

The aim of this study was to assess levels of IL-17A, IL-17E/IL-25, IL-17F, and TNF-*α* in the saliva in patients with oral and oropharyngeal cancer, depending on the degree of malignancy and bacteriological cultures from the oral cavity as potential biomarkers of cancer.

## 2. Material and Methods

### 2.1. Patient Characteristics

The study included 71 patients subjected to screening tests with histopathological diagnosis of oral and/or oropharyngeal cancer hospitalized in the Clinical Oncology Clinic and the Maxillofacial Surgery Clinic of Fryderyk Chopin Provincial Specialist Hospital No. 1 in Rzeszów, Poland. In the studied group, the majority of patients were male (78.9%). The age of the respondents ranged from 45 to 78 years of age (mean age 60.00 ± 7.88). 91.5% of the patients reported smoking, and 52.1% indicated alcohol abuse. The neoplastic lesions were located in the oral cavity bed (*n* = 5), oropharynx (*n* = 4), palatine tonsil (*n* = 18), palate (*n* = 7), tongue (*n* = 21), and other locations (*n* = 16). Clinical stage was determined on the basis of imaging tests (computed tomography and ultrasound) using the TNM classification from 2012. Three groups were distinguished in the studied group according to the clinical stage. The first group patients were stage I (*n* = 1)+II (*n* = 13), the second stage III (*n* = 10), and the third group included patients with stage IV (*n* = 47). Due to the small number of patients with stage I, the first group was combined in patients qualified for surgery. Histopathological evaluation revealed 24 patients with a good differentiated (G1) tumor, 29 patients with an intermediate grade (G2), and 13 patients with a poor grade of histopathological differentiation (G3). In 5 patients, the grade was not determined. The procedure was conducted before the start of surgical treatment or chemotherapy. In addition, oral swabs for microbiological culture were collected from the primary lesion and the throat including standard laboratory tests. The biological samples were collected noninvasively, with no adverse effects on the patients. Sampling and data processing were carried out in accordance with the Helsinki Declaration and all participants signed an informed consent. The study was approved by the Bioethics Committee of the University of Rzeszów (approval No. 1/02/2016).  Table 1 presents clinical and demographic characteristics of the patients included in this study.

### 2.2. Saliva Sampling

Saliva samples were collected from each subject in the morning. Approximately 5 mL of saliva was collected from each individual patient. The patients were asked to avoid drinking, eating, smoking, and using oral hygiene products for at least 1 hour before saliva sampling. Samples were centrifuged at 1000 × g and +4°C. The supernatant was collected, aliquoted, and cryopreserved at -86°C until the measurement.

### 2.3. Estimation of Salivary Cytokine Concentration

The salivary concentrations of IL-17A, IL-17E/IL-25, and IL-17F were evaluated with the use of commercially available ELISA kits (Diaclone, France and Cloud-Clone Corp, China). Measurements and analysis were performed with the use of Tecan Infinite M200 plate reader (Tecan Group Ltd., Switzerland) and Magellan™ software (Tecan Group Ltd., Switzerland). The salivary concentration of TNF-*α* was measured with the use of a FLEXMAP 3D® multiplexing platform (Merck Millipore, USA) and Luminex kit for human TNF-*α* (Merck Millipore, USA). All procedures were performed according to the manufacturer's protocols.

### 2.4. Bacterial Cultures of Oral Swabs

Oral swabs were done from the surface of the tumor. Bacterial cultures were performed in the Laboratory of Bacteriology of Medical Diagnostic Laboratory of Fryderyk Chopin Provincial Specialist Hospital No. 1 in Rzeszów, Poland. Columbia Agar with 5% sheep blood, CHROMID® CPS® Elite, CHROMID® MRSA, and CHROMID® Candida (all of them bioMérieux SA, France) solid media were used for identification of aerobic bacteria and candida. Cultures for identification of bacteria were incubated for 24 h at 37°C and for candida culture for 48 h at 35°C.For isolation of anaerobic bacteria Schaedler agar +5% sheep blood (bioMérieux SA, France) were used and was incubated for 48 h in GENBAG ANAEROBIC sachets (bioMérieux SA, France) in an incubator at 37°C. Bacteria and yeasts were identified with the use of VITEK® 2 cards (bioMérieux SA, France) according to the manufacturer's protocol.

### 2.5. Statistical Analysis

Statistical analyses were performed using Statistica 13.1 PL (StatSoft, Poland) and GraphPad Prism 8.0.0 (GraphPad Software Inc., USA). The Kolmogorov-Smirnov test and Lilliefors test were used to assess the distribution of variables; due to non-normal distribution, nonparametric tests were used for detailed analysis. The quantitative variables are presented as the median value and minimum and maximum values. The Mann–Whitney *U* test was used for comparisons between 2 groups, and the Kruskal-Wallis test and Dunn's multiple comparison post hoc test were used to compare ≥3 groups. *p* < 0.05 was considered to be an indication of statistical significance.

## 3. Results

### 3.1. Analysis of Association of Salivary Cytokine Concentration and Disease Stage

The concentrations of IL-17A, IL-17F, IL-17E/IL-25, and TNF-*α* in saliva samples in the patients diagnosed with oral and oropharyngeal cancer was correlated with the disease stage as well as with T, N, and M parameters separately.

**Figure 1:**
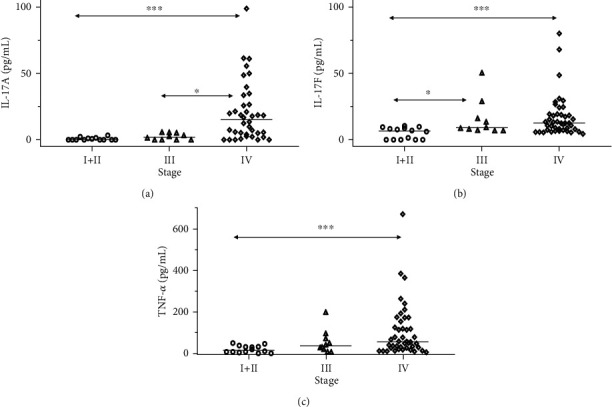
Differences in concentrations of IL-17A, IL-17F, and TNF-*α* between patients with head and neck cancer at different stages of disease. (a) There was a significant difference in salivary concentration of IL-17A (*p* < 0.001) between patients with different disease stage, and post hoc tests showed significant differences between stage I/II patients as well as stage III and stage IV patients. (b) There was a significant difference in salivary concentration of IL-17F (*p* < 0.01) between stages, and post hoc tests showed significant differences between stage I/II patients as well as stage IV patients and stage III patients. (c) There was a significant difference in salivary concentration of TNF-*α* (*p* < 0.01) between stages, and post hoc tests showed significant differences between stage I/II patients and stage IV patients. ^∗^*p* < 0.05, ^∗∗∗^*p* < 0.001, _median.

With the use of the Kruskal-Wallis ANOVA test, we found significantly higher salivary levels of IL-17A (*p* < 0.001), IL-17F (*p* < 0.01), and TNF-*α* (*p* < 0.01) in patients with more advanced disease (Figure 1). We did not find any significant difference in salivary concentration of IL-17E/IL-25 between patients with different stages of disease. The descriptive statistics and detailed results of the statistical analysis are shown in Supplementary Table I. We noticed that salivary concentrations of IL-17A, IL-17F, and TNF-*α* were highest in stage IV patients and lowest in stage I or II patients.

**Figure 2:**
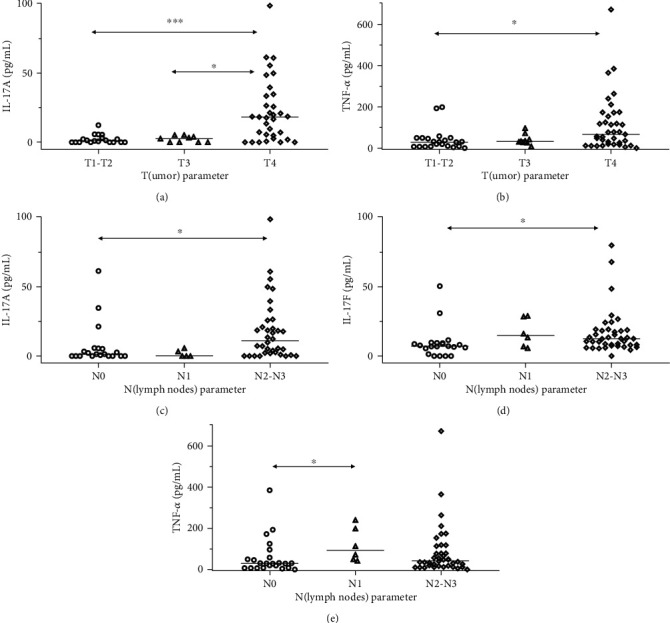
Differences in concentrations of IL-17A, IL-17F, and TNF-*α* between patients with head and neck cancers depending on T or N parameters. (a) There was a significant difference in salivary concentration of IL-17A (*p* < 0.001) between patients with different sizes of primary tumor; post-hoc tests showed significant differences between stage T4 patients and T1-T2 as well as T3 patients. (b) There was a significant difference in salivary concentration of TNF-*α* (*p* < 0.05) between stages and post hoc tests showed significant differences between stage T4 patients and T1-T2 patients. (c) There was a significant difference in salivary concentration of IL-17A (*p* < 0.01) between stages and post hoc tests showed significant differences between N2-N3 patients and N0 patients. (d) There was a significant difference in salivary concentration of IL-17F (*p* < 0.05) between stages, and post hoc tests showed significant differences between N2-N3 patients and N0 patients. (e) There was a significant difference in salivary concentration of TNF-*α* (*p* < 0.05) between stages, and post hoc tests showed significant differences between N1 patients and N0 patients. ^∗^*p* < 0.05, ^∗∗∗^*p* < 0.001, _median.

We also found that patients with larger primary tumors (T4) were characterized by significantly higher salivary concentrations of IL-17A (Figure 2(a)) and TNF-*α* (Figure 2(b)). The more advanced involvement of lymph nodes was also characterized by significantly higher levels of IL-17A (Figure 2(c)) and IL-17F (Figure 2(d)) in the saliva. The patients with N0 parameter showed the lowest level of salivary TNF-*α*, and this difference was statistically significant when compared to patients with N1, but not between N0 and N2-N3 patients (Figure 2(e)). Descriptive statistics and *p* values are shown in Supplementary Table II and Table III.

### 3.2. Analysis of Association between Salivary Cytokine Concentrations and Grade of Malignancy

We did not find any statistically significant differences in the concentration of TNF-*α*, IL-17A, IL-17E/IL-25, and IL-17F depending on the grade of malignancy.

### 3.3. Analysis of Association of Salivary Cytokine Concentrations with Bacterial Culture of Oral Swabs

**Figure 3:**
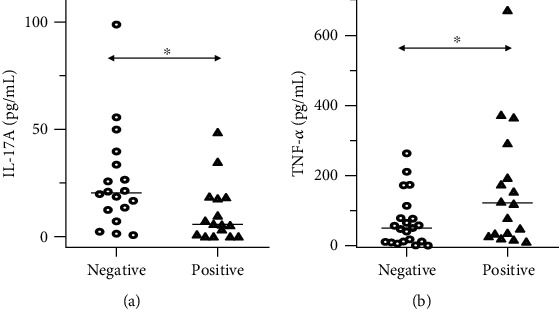
Differences in concentrations of IL-17A and TNF-*α* between patients with head and neck cancers depending on results of oral swab bacterial culture. (a) There was a significant difference in salivary concentration of IL-17A (*p* < 0.05) between patients with negative and positive aerobic bacterial cultures. (b) There was a significant difference in salivary concentration of TNF-*α* (*p* < 0.05) between patients with negative and positive aerobic bacterial cultures. ^∗^*p* < 0.05, _median.

A lower level of IL-17A concentration was found in the case of positive aerobic culture and statistical significance was noted. A significantly (*p* = 0.01) higher level of IL-17A was found in patients with negative aerobic culture of oral swab (Figure 3(a)). On the other hand, the concentration of TNF-*α* was significantly (*p* = 0.04) lower in patients with negative oral aerobic bacterial culture (Figure 3(b)). Detailed results of this analysis are summarized in Supplementary Table IV. We did not find any statistically significant differences between patients with positive or negative results of anaerobic bacterial cultures. On the other hand, salivary concentration of IL-17F was almost twice higher in patients with negative anaerobic bacterial cultures of oral swabs and *p* = 0.06.

### 3.4. Analysis of Association of Salivary Cytokine Concentration with Alcohol Consumption, Cigarette Smoking, and Patient Inhabitancy

The study assessed the effects of alcohol consumption, smoking, and patient residence on cytokine concentration. Alcohol consumption and smoking did not influence the levels of tested cytokines. The influence of place of residence on the average level of cytokine concentration did not reveal any statistically significant differences.

### 3.5. Correlations between Salivary Concentration of Examined Cytokines

We found positive statistically significant correlation between salivary concentrations of IL-17A and IL-17F in the group of all patients. In the group of patients with different stages of disease, there was one significant correlation between concentrations of IL-17A and TNF-*α* in the group of stage I+II patients.

## 4. Discussion

In order to improve early diagnosis and reduce mortality, there is a need to develop markers with an appropriate level of sensitivity and specificity. According to the available literature, many potential markers have been identified depending on the region of the world where the study was conducted.

TNF-*α* is suggested to be a marker of oral squamous cell carcinoma, and its concentration has been showed to be higher in the saliva than in plasma due to high local cytokine production [11, 13, 22]. Higher levels of TNF-*α* in the saliva compared to serum may indicate cytokine activity directly in the tumor environment [23]. Increased levels of TNF-*α* in both the saliva and serum in patients with oral squamous cell carcinoma compared to a healthy control group have been reported [23]. On the other hand, Sahebjamee et al. did not find statistically significant elevated levels of TNF-*α* in the saliva of patients with oral squamous cell carcinoma [24]. In our study, as in other works, an increase in TNF-*α* concentration in clinical stage IV was observed, and in contrast to other studies, there was no increase in the level of this cytokine in poor and intermediate differentiated tumors compared to good differentiated lesions [23, 24]. Polz-Dacewicz et al. also determined the relationship of TNF-*α* levels and T in their study and found a relationship between TNF-*α* concentration and increasing tumor size [25]. The increase in TNF-*α* concentration correlated with higher T and N parameters was also confirmed by our study. Excessive TNF-*α* concentration in the tumor microenvironment shifts the balance towards invasion, resulting in a reduction in overall survival and disease-free survival [26]. Our study also showed an increase in TNF-*α* concentration in the saliva of patients with positive aerobic bacterial culture of oral swabs. A review of the literature did not reveal any studies assessing TNF-*α* levels depending on bacteriological culture.

Th17 cells and IL-17 levels are reported to be increased in the peripheral blood of patients with squamous cell carcinoma of the head and neck [21]. Wei et al. found that an increased level of IL-17A in the tumor microenvironment affects the acceleration of the progression of SCC of the tongue [27]. When IL-17 levels in the serum and tissues were determined, IL-17A levels were higher in patients with lymph node metastases or advanced clinical status and no significant differences in histological assessment and tumor size were found [27]. In our study, we found significant relationships between the concentration of IL-17A and stage IV as well as higher values of T and N parameters considered separately. Our results are in accordance with other studies and, taken together, could suggest that IL-17A plays a potential role in carcinogenesis and progression of oral and oropharyngeal cancers, and elevated levels of IL-17A in saliva may be a new biomarker for predicting the aggressive phenotype of an oral tumor.

IL-17F has been detected in various cancers. Elevated IL-17F concentration occurs, i.e., in prostate cancer [28]. In a study by Ding et al., serum IL-17F and VEGF levels were assessed in healthy subjects, subjects with leukoplakia, and in subjects with OSCC. It was observed that IL-17F levels were elevated in healthy individuals compared to the OSCC group [29]. To the best of our knowledge, our study is the first showing an association of salivary IL-17 with OSSC. We showed that the difference in IL-17F level was statistically significant between groups with stage I, II, and IV and between stage I, II, and III. The difference in IL-17F level was statistically significantly and associated with involvement of lymph nodes.

The positive correlation in patients with lower stages could be associated with inflammation caused by other factors than cancer and present before neoplastic process. In more advanced patient, inflammation seems to be associated with chronic process involving Th17 lymphocytes and relatively more intensive production of IL-17A and IL-17F than TNF-*α* produced by innate immune cells. The chronic inflammation associated with tumor and ulceration could be also associated with significantly higher of IL-17A in patients with negative aerobic bacterial cultures of oral swabs; also, IL-17F concentration was higher in patients with negative anaerobic bacterial cultures of oral swabs, but this difference was not statistically significant. The higher salivary concentration of TNF-*α* in patients with positive aerobic bacterial cultures could be associated with innate immune response.

To our surprise, we did not find any significant correlations between concentration of examined proinflammatory cytokines and cigarette smoking, although this is in accordance with other studies [7, 13, 23].

The possible limitation of our study is lack of control group of healthy individuals. On the other hand, the control group shall be carefully matched not only for sex and age but also for hygiene status of oral cavity, e.g., number of carious teeth.

## 5. Conclusions

We found that increased salivary concentration of IL-17A, IL-17F, and TNF-*α* is associated with advanced oral squamous cell carcinoma. IL-17A seems to be an especially promising biomarker because its presence is significantly correlated with stage of disease as well as with primary tumor size with lymph node involvement being considered separately. In contrast to TNF-*α*, an increased concentration of IL-17A is not associated with the presence of bacteria on neoplastic lesions. The use of saliva makes estimation of IL-17A simple and safe. It is necessary to conduct further tests to confirm the usefulness of estimation of IL-17A in saliva in the diagnosis, clinical prognosis, and monitoring of the effects of treatment of oral cavity cancer.

## Figures and Tables

**Table 1:** Characteristics of the study group.

		*n*	%
Sex	Female	15	21.1
	Male	56	78.9
Age (years)	<50	10	14
	≥50	61	86
Place of residence	Urban	39	54.9
	Rural	32	45.1
Smoking	Yes	65	91.5
	No	6	8.5
Excessive use of alcohol	Yes	37	52.1
	No	34	47.9
Histological tumor type	Squamous cell carcinoma	66	92.9
	Other	5	7.1
Histological grade	G1	24	33.8
	G2	29	40.8
	G3	13	18.3
	Not determined	5	7.1
Lesion location	Oral cavity	5	7.0
	Oropharynx	4	5.6
	Palatine tonsil	18	25.3
	Palate	7	9.8
	Tongue	21	29.5
	Other	16	22.8
Clinical staging	I	1	1.4
	II	13	18.3
	III	10	14.1
	IV	47	66.2

## Data Availability

The data used to support the findings of this study are available from the corresponding author upon request.

## Supplementary Materials

Supplementary Materials Supplementary Table I: analysis of TNF-*α*, IL-17A, IL-17E, and IL-17F concentrations according to disease stage. Supplementary Table II: statistically significant relationships between cytokine levels and T (size or direct extent of the primary tumor) parameter. Supplementary Table III: statistically significant relationships between cytokine levels and N (degree of spread to regional lymph nodes) parameter. Supplementary Table IV: relationship between the levels of cytokines tested and the results of bacteriological cultures from the oral cavity. (1) Aerobic culture. (2) Anaerobic culture. (+) Positive. (-) Negative. Supplementary Table V: correlations between salivary concentration of examined cytokines.

## References

[1] Bray F., Ferlay J., Soerjomataram I., Siegel R. L., Torre L. A., Jemal A. (2018). Global cancer statistics 2018: GLOBOCAN estimates of incidence and mortality worldwide for 36 cancers in 185 countries. *CA: a Cancer Journal for Clinicians*.

[2] Bray F., Ferlay J., Soer’jomataram I., Siegel R. L., Torre L. A., Jemal A. (2020). Incidence trends of lip, oral cavity, and pharyngeal cancers: global burden of disease 1990–2017. *Journal of Dental Research*.

[3] Cheng Y.-S. L., Wright J. (2011). Advances in diagnostic adjuncts for oral squamous cell carcinoma. *The Open Pathology Journal*.

[4] Elashoff D., Zhou H., Reiss J. (2012). Prevalidation of salivary biomarkers for oral cancer detection. *Cancer Epidemiol Biomarkers Prev*.

[5] Arellano-Garcia M. E., Hu S., Wang J. (2008). Multiplexed immunobead-based assay for detection of oral cancer protein biomarkers in saliva. *Oral Diseases*.

[6] Brinkmann O., Kastratovic D. A., Dimitrijevic M. V. (2011). Oral squamous cell carcinoma detection by salivary biomarkers in a Serbian population. *Oral Oncology*.

[7] Juretić M., Cerović R., Belušić-Gobić M. (2013). Salivary levels of TNF-*α* and IL-6 in patients with oral premalignant and malignant lesions. *Folia biologica*.

[8] Brailo V., Vucicevic-Boras V., Lukac J. (2012). Salivary and serum interleukin 1 beta, interleukin 6 and tumor necrosis factor alpha in patients with leukoplakia and oral cancer. *Medicina Oral, Patología Oral y Cirugía Bucal*.

[9] Hohberger L., Wuertz B. R. K., Xie H., Griffin T., Ondrey F. (2008). TNF-alpha drives matrix metalloproteinase-9 in squamous oral carcinogenesis. *The Laryngoscope*.

[10] Hoffmann T. K., Sonkoly E., Homey B. (2007). Aberrant cytokine expression in serum of patients with adenoid cystic carcinoma and squamous cell carcinoma of the head and neck. *Head & Neck*.

[11] Nakano Y., Kobayashi W., Sugai S., Kimura H., Yagihashi S. (1999). Expression of tumor necrosis factor-alpha and interleukin-6 in oral squamous cell carcinoma. *Japanese Journal of Cancer Research*.

[12] Korostoff A., Reder L., Masood R., Sinha U. K. (2011). The role of salivary cytokine biomarkers in tongue cancer invasion and mortality. *Oral Oncology*.

[13] Rhodus N. L., Ho V., Miller C. S., Myers S., Ondrey F. (2005). NF-kappaB dependent cytokine levels in saliva of patients with oral preneoplastic lesions and oral squamous cell carcinoma. *Cancer Detection and Prevention*.

[14] Zou W., Restifo N. P. (2010). T(H)17 cells in tumour immunity and immunotherapy. *Nature Reviews Immunology*.

[15] Kato T., Furumoto H., Ogura T. (2001). Expression of IL-17 mRNA in ovarian cancer. *Biochemical and Biophysical Research Communications*.

[16] Li M., Feurino L. W., Li F. (2007). Thymosinalpha1 stimulates cell proliferation by activating ERK1/2, JNK, and increasing cytokine secretion in human pancreatic cancer cells. *Cancer Letters*.

[17] Gu F. M., Li Q. L., Gao Q. (2011). IL-17 induces AKT-dependent IL-6/JAK2/STAT3 activation and tumor progression in hepatocellular carcinoma. *Molecular Cancer*.

[18] Numasaki M., Fukushi J.-i., Ono M. (2003). Interleukin-17 promotes angiogenesis and tumor growth. *Blood*.

[19] Lan C., Huang X., Lin S. (2013). High density of IL-17-producing cells is associated with improved prognosis for advanced epithelial ovarian cancer. *Cell and Tissue Research*.

[20] Benchetrit F., Ciree A., Vives V. (2002). Interleukin-17 inhibits tumor cell growth by means of a T-cell-dependent mechanism. *Blood*.

[21] Li C., Zhao Y., Zhang W., Zhang W. (2011). Increased prevalence of T_H_17 cells in the peripheral blood of patients with head and neck squamous cell carcinoma. *Oral Surgery, Oral Medicine, Oral Pathology, Oral Radiology, and Endodontics*.

[22] Rhodus N. L., Cheng B., Myers S., Miller L., Ho V., Ondrey F. (2005). The feasibility of monitoring NF‐*κ*B associated cytokines: TNF‐*α*, IL‐1*α*, IL‐6, and IL‐8 in whole saliva for the malignant transformation of oral lichen planus. *Molecular carcinogenesis*.

[23] Krishnan R., Thayalan D. K., Padmanaban R., Ramadas R., Annasamy R. K., Anandan N. (2014). Association of serum and salivary tumor necrosis factor-*α* with histological grading in oral cancer and its role in differentiating premalignant and malignant oral disease. *Asian Pacific Journal of Cancer Prevention*.

[24] SahebJamee M., Eslami M., AtarbashiMoghadam F., Sarafnejad A. (2008). Salivary concentration of TNF*α*, IL1*α*, IL6, and IL8 in oral squamous cell carcinoma. *Medicina Oral, Patología Oral y Cirugía Bucal*.

[25] Polz-Dacewicz M., Strycharz-Dudziak M., Dworzański J., Stec A., Kocot J. (2016). Salivary and serum IL-10, TNF-*α*, TGF-*β*, VEGF levels in oropharyngeal squamous cell carcinoma and correlation with HPV and EBV infections. *Infectious Agents and Cancer*.

[26] Goertzen C., Mahdi H., Laliberte C. (2018). Oral inflammation promotes oral squamous cell carcinoma invasion. *Oncotarget*.

[27] Wei T., Cong X., Wang X. T. (2017). Interleukin-17A promotes tongue squamous cell carcinoma metastasis through activating miR-23b/versican pathway. *Oncotarget*.

[28] Liu Y., Zhao X., Sun X. (2015). Expression of IL-17A, E, and F and their receptors in human prostatic cancer: comparison with benign prostatic hyperplasia. *The Prostate*.

[29] Ding L., Hu E. L., Xu Y. J. (2015). Serum IL-17F combined with VEGF as potential diagnostic biomarkers for oral squamous cell carcinoma. *Tumour Biology*.

