# Supplementary material for: Salivary IL-17A, IL-17F, and TNF-α Are Associated with Disease Advancement in Patients with Oral and Oropharyngeal Cancer
Source: J Immunol Res. 2020 Aug 13;2020:3928504. doi: 10.1155/2020/3928504 (PMC7443019; doi:10.1155/2020/3928504)
Supplement: Supplementary Materials — Supplementary Table I: analysis of TNF-α, IL-17A, IL-17E, and IL-17F concentrations according to disease stage. Supplementary Table II: statistically significant relationships between cytokine levels and T (size or direct extent of the primary tumor) parameter. Supplementary Table III: statistically significant relationships between cytokine levels and N (degree of spread to regional lymph nodes) parameter. Supplementary Table IV: relationship between the levels of cytokines tested and the results of bacteriological cultures from the oral cavity. (1) Aerobic culture. (2) Anaerobic culture. (+) Positive. (-) Negative. Supplementary Table V: correlations between salivary concentration of examined cytokines. [file 3928504.f1.pdf]

Supplementary Table I. Analysis of TNF-α, IL-17A, IL-17E, IL-17F concentrations according to disease stage.

| | | Median | Min | Max | p Kruskal-Wallis ANOVA |
|---|---|---|---|---|---|
| IL-17A (pg/ml) | I-II | 0.00 | 0.00 | 3.30 | <0.001 |
| | III | 2.02 | 0.00 | 5.69 | |
| | IV | 15.11 | 0.00 | 98.85 | |
| IL-17E (ng/ml) | I-II | 0.07 | 0.00 | 0.09 | 0.25 |
| | III | 0.07 | 0.00 | 0.07 | |
| | IV | 0.075 | 0.00 | 0.08 | |
| IL 17F (ng/ml) | I-II | 0.006 | 0.00 | 0.01 | <0.01 |
| | III | 0.009 | 0.01 | 0.05 | |
| | IV | 0.012 | 0.00 | 0.08 | |
| TNF-α (pg/ml) | I-II | 14.65 | 0.00 | 49.47 | <0.01 |
| | III | 36.35 | 6.53 | 198.67 | |
| | IV | 55.62 | 5.48 | 672.07 | |

Supplementary Table II. Statistically significant relationships between cytokine levels and T (size or direct extent of the primary tumor) parameter.

| | | Median | Min | Max | p Kruskal-Wallis ANOVA |
|---|---|---|---|---|---|
| | T1-T2 | 1.00 | 0.00 | 12.42 | <0.001 |

| | | | | | |
|---|---|---|---|---|---|
| IL-17A (pg/ml) | T3 | 2.62 | 0.00 | 5.23 | |
| | T4 | 18.30 | 0.00 | 98.85 | |
| TNF-α (pg/ml) | T1-T2 | 29.24 | 0.00 | 198.67 | <0.05 |
| | T3 | 33.39 | 7.95 | 96.09 | |
| | T4 | 66.84 | 0.00 | 672.07 | |

Supplementary Table III. Statistically significant relationships between cytokine levels and N (degree of spread to regional lymph nodes) parameter.

| | | Median | Min | Max | P Kruskal-Wallis ANOVA |
|---|---|---|---|---|---|
| IL-17A (pg/ml) | N0 | 1.41 | 0.00 | 61.44 | <0.01 |
| | N1 | 0.00 | 0.00 | 5.69 | |
| | N2-N3 | 11.10 | 0.00 | 98.85 | |
| IL 17F (ng/ml) | N0 | 0.007 | 0.00 | 0.05 | <0.05 |
| | N1 | 0.015 | 0.005 | 0.02 | |
| | N2-N3 | 0.012 | 0.00 | 0.08 | |
| TNF-α (pg/ml) | N0 | 29.81 | 0.00 | 385.18 | <0.05 |
| | N1 | 92.77 | 42.01 | 240.13 | |
| | N2-N3 | 40.04 | 0.00 | 672.07 | |

Supplementary Table IV. Relationship between the levels of cytokines tested and the results of bacteriological cultures from the oral cavity. (1) Aerobic culture, (2) Anaerobic culture, (+) positive, (-) negative.

| | | | Median | Min | Max | p |
|---|---|---|---|---|---|---|
| IL-17A (pg/ml) | 1 | + | 5.69 | 0.00 | 48.60 | 0.01 |
| | | - | 20.40 | 0.71 | 98.85 | |
| | 2 | + | 15.91 | 0.71 | 98.85 | 0.35 |
| | | - | 13.25 | 0.00 | 55.60 | |
| IL-17E (ng/ml) | 1 | + | 0.07 | 0.00 | 0.10 | 0.67 |
| | | - | 0.07 | 0.00 | 0.08 | |
| | 2 | + | 0.07 | 0.07 | 0.08 | 0.31 |
| | | - | 0.07 | 0.00 | 0.10 | |
| IL 17F (ng/ml) | 1 | + | 0.01 | 0.00 | 0.10 | 0.93 |
| | | - | 0.01 | 0.00 | 0.06 | |
| | 2 | + | 0.01 | 0.00 | 0.02 | 0.06 |
| | | - | 0.01 | 0.00 | 0.10 | |
| TNF-α (pg/ml) | 1 | + | 121.77 | 10.92 | 1179.80 | 0.04 |
| | | - | 50.09 | 0.00 | 263.49 | |
| | 2 | + | 27.86 | 0.00 | 1179.76 | 0.42 |
| | | - | 64.92 | 0.28 | 372.70 | |

Supplementary Table V. Correlations between salivary concentration of examined cytokines.

| All patients | | | | |
|---|---|---|---|---|
| | IL-17A (pg/ml) | IL-17E (ng/ml) | IL 17F (ng/ml) | TNF-α (pg/ml) |
| IL-17A (pg/ml) | - | R=0.17 NS | **R=0.29 p=0.03** | R=0.17 NS |
| IL-17E (ng/ml) | - | - | R=-0.15 NS | R=-0.05 NS |
| IL 17F (ng/ml) | - | - | - | R=-0.007 NS |
| Stage I+II | | | | |
| IL-17A (pg/ml) | - | R=-0.03 NS | R=0.36 NS | **R=0.67 p=0.02** |
| IL-17E (ng/ml) | - | - | R=-0.48 NS | R=0.09 NS |
| IL 17F (ng/ml) | - | - | - | R=-0.14 NS |
| Stage III | | | | |

| | | | | |
|---|---|---|---|---|
| IL-17A (pg/ml) | - | R=-0.10 NS | R=0.15 NS | R=-0.22 NS |
| IL-17E (ng/ml) | - | - | R=-0.07 NS | R=-0.19 NS |
| IL 17F (ng/ml) | - | - | - | R=-0.04 NS |
| Stage IV | | | | |
| IL-17A (pg/ml) | - | R=0.04 NS | R=-0.07 NS | R=0.05 NS |
| IL-17E (ng/ml) | - | - | R=-0.20 NS | R=-0.11 NS |
| IL 17F (ng/ml) | - | - | - | R=0.01 NS |
